# A Systematic Investigation of Tobacco Industry Sourced Data Relating to Illicit Tobacco Trade Featured in Pakistan’s Media Coverage (2015–2020)

**DOI:** 10.1093/ntr/ntae133

**Published:** 2024-05-31

**Authors:** Allen William Andrew Gallagher, Zaineb Danish Sheikh, Zohaib Khan, Urooj Aftab, Mariyam Rahim, Asad Ullah, Safat Ullah, Hessam Ul Haq, Kamran Siddiqi

**Affiliations:** Tobacco Control Research Group (TCRG), University of Bath, UK; Tobacco Control Research Group (TCRG), University of Bath, UK; Institute of Public Health & Social Sciences, Khyber Medical University, Peshawar, Pakistan; Institute of Public Health & Social Sciences, Khyber Medical University, Peshawar, Pakistan; Institute of Public Health & Social Sciences, Khyber Medical University, Peshawar, Pakistan; Institute of Public Health & Social Sciences, Khyber Medical University, Peshawar, Pakistan; Institute of Public Health & Social Sciences, Khyber Medical University, Peshawar, Pakistan; Institute of Public Health & Social Sciences, Khyber Medical University, Peshawar, Pakistan; Department of Health Sciences, University of York, York, UK; Hull York Medical School, University of York, UK

## Abstract

**Introduction:**

Previous evidence suggests the tobacco industry uses media to disseminate misleading narratives relating to illicit tobacco trade (ITT) as part of efforts to influence policy outcomes. Such evidence is largely high-income countries (HIC) focused, resulting in a literature gap for low- and middle-income countries (LMICs). Pakistan and its annual budget cycle are used as a case study for addressing this gap.

**Aims and Methods:**

Electronic English-language articles from newspapers in Pakistan (328) were sourced from LexisNexis and a sub-sample of Urdu-language electronic articles (12) were identified through internet searches. The articles were published between 2015 and 2020 and included claims/estimates relating to ITT, which were coded to identify cited data sources. Changes in media coverage before and after Pakistan’s annual budget announcements were explored via Wilcoxon signed rank and Poisson regression tests.

**Results:**

Of the 357 claims/estimates analyzed, 66 (20%) were industry-funded. The most prevalent sources were national government bodies (36.6%) and tobacco companies or their representatives (15.1%). Wilcoxon signed-rank and Poisson regression tests on the frequency of English-language articles both created a *p*-value of < .05 for the frequency of relevant articles between the months of April and May, compared to the other months, indicating statistical significance.

**Conclusions:**

There was a statistically significant increase in the number of English-language articles featuring claims/estimates relating to Pakistan’s ITT in the months leading up to the annual budget each year. The government should consider measures to improve transparency standards within media coverage and promote factcheck journalism to safeguard against industry tactics to manipulate public discourses.

**Implications:**

This paper is, to the best of our knowledge, the largest exploration of the use of data sourced from the tobacco industry within a country’s media that has been undertaken to date, utilizing a team of seven coders across the United Kingdom and Pakistan. Our findings reveal weaknesses within media coverage of ITT in Pakistan, both in English and Urdu language publications. We encourage the government to consider new standards to enhance transparency and promote factcheck journalism within media coverage in the country.

## Introduction

Illicit tobacco trade (ITT) has widespread negative public health and economic impacts. By increasing the accessibility and affordability of tobacco products, ITT undermines the effectiveness of tobacco control policies in reducing smoking prevalence. This is particularly true for the most price-sensitive populations such as those with lower incomes, including underage smokers.^1^ Its economic impacts include increasing tobacco-related morbidity and mortality costs^2^ as well as considerable loss of productivity, government taxes, and duty revenue.^3^

Adding to these problems is that the illegal nature of the trade makes it intrinsically difficult to measure, and thus also to effectively address.^4^ Owing to the complexities of data collection on ITT, there is a lack of Government and independently produced data on the topic in many countries, rendering the tobacco industry as often the most prominent data source on ITT in countries throughout the world.^5,6^

According to the World Health Organization (WHO)^7^ and estimates of the potential impact of eliminating the practice,^3,8^ the burden of ITT falls disproportionately on low and middle-income countries (LMICs), where the majority of the world’s tobacco users live. One such LMIC is Pakistan, the focus country of our study, which signed the WHO Framework Convention on Tobacco Control (FCTC) in 2005 and ratified the FCTC Protocol to Eliminate Illicit Trade in Tobacco Products (The Protocol) in 2018. In Pakistan, the responsibility to restrict ITT lies largely with the Federal Board of Revenue which is responsible for the country’s tobacco track and trace system (mandated in 2022) and produces regular updates of raids and seizures of ITT on its website. In 2019, the Inland Revenue Enforcement Network was established to combat ITT and to ensure the use of the track and trace system, once implemented.^9^

In recent years, numerous reports commissioned or conducted by tobacco companies (as well as the Philip Morris-funded Foundation for Smoke-Free World)^10^ have provided estimates of Pakistan’s ITT. Several of these reports such as the “Asia Illicit Tobacco Indicator” series, conducted by Oxford Economics^11^ and the International Tax & Investment Center (OE & ITIC),^12^ have been criticized in academic literature for their methodologies, bringing into question the validity of their estimates.^10,13^ A recent independent study on ITT in Pakistan found its estimates to be “considerably lower” than those presented by the industry, referring also to this being a trend in the literature when estimates from sources not affiliated with the industry are compared with those from industry.^14^ These discrepancies between independent and industry-affiliated estimates suggest that TI estimates of Pakistan’s ITT may be exaggerated, in line with previous findings from other countries.^10,15–17^

It is well-documented that TI-generated estimates can be influential in deterring governments from imposing higher taxes by stoking fears of illicit trade, regardless of their accuracy.^18–20^ There is precedent for this within Pakistan where, in 2019, the Federal Board of Revenue (a national tax collection agency) withdrew its decision to increase tax rates on the basis of an industry-funded report, which estimated the illicit trade at 41%.^14^

Such estimates can also be propagated by media outlets. Previous empirical evidence suggests that the industry has been using media to construct alternative narratives and to mislead policymakers and the public by promoting its self-funded reports.^21,22^ Most of the evidence on the industry’s use of media campaigns to spread favorable narratives about ITT comes from high-income countries (HICs) such as the United Kingdom,^23,24^ although some specific examples have been identified in other countries too.^25^ Ultimately, there continues to be a significant gap in literature on the topic of TI manipulation of media within an LMIC context, including in Pakistan specifically. This is problematic as HIC findings are not always transferrable to LMIC settings due to a number of factors, for example, differences in the tobacco market, industry’s behavior/strategies, information disparities, and differing socio-cultural and political factors.^26^

This study aims to address this gap by systematically reviewing the proportion of TI-sourced claims regarding ITT that were featured in media coverage in Pakistan throughout 2015–2020 and exploring if there were any notable changes in the frequency of such instances in the lead-up to and directly following the announcement of the annual budget for each included year. We also explore the nature of claims and estimates within such articles, for instance policy recommendations identified throughout the dataset (Supplementary Appendix). As such, our study does not focus on producing independent ITT estimates for Pakistan, as this has been done already,^14^ but instead focuses on assessing the frequency, timing, nature of, and sources of origin of ITT claims and estimates cited in newspapers in Pakistan.

## Materials and Methods

### Systematic Approach

We approached data collection and analysis for this work in line with the PRISMA guidelines for a systematic review, although we did not formally register our study as a review in PROSPERO nor produce a formal review protocol due to our study’s focus on newspaper articles rather than more conventional data sources for systematic reviews for example, academic studies/clinical trials. As such, we developed the paper following the PRISMA checklist for systematic reviews, though not all aspects of the list were relevant to the nature of our study.

### Data Search and Inclusion Criteria

Electronic newspaper articles, sourced from the LexisNexis media database, were collected in November 2022 using the following search strand:

“Pakistan” AND (“illegal” OR “illicit” OR “smuggl*) AND (“tobacco” OR “cigar*) AND (‘British American Tobacco’ OR Imperial Tobacco” OR “Japan Tobacco” OR “Pakistan Tobacco Company” OR--) “Philip Morris International”)

An initial broader strand which did not feature the names of specific tobacco companies was employed; however, this produced a substantially larger number of results that it would not have been practical to code, even with such a large coding team.

To ensure that collected articles met our inclusion criteria, results were filtered accordingly (Box 1) using LexisNexis’ filtering tool. Our reasoning for focusing on 2015–2020 was to capture the media landscape prior to and following Pakistan’s ratification of the Protocol in 2018.

**Box 1. Inclusion Criteria and Key Definitions**

**Stage 1 (Conducted Via Search Filtering)**

English-language newspaper articles, relevant to the topic of Pakistan’s illicit tobacco trade and published between 2015 and 2020.

Article must provide a claim and/or estimate about Pakistan’s ITT and must not be a duplicate.

**Stage 2 (Conducted Manually on Articles Identified From Search)**

**Definitions**

Illicit tobacco trade = reference to the production, import, export, purchase, sale, or possession of tobacco goods which fail to comply with the national legislation. In addition to capturing products where full duties have not been paid, this definition also captures products which are duty-paid but are in breach of other national legislation, for example, health warning requirements.

Claims/estimates = This includes quantitative data/an estimate of illicit tobacco trade in Pakistan (an approximate calculation or judgment related to illicit trade, for example, % of market that is illicit, number of Rupees lost as a result of illicit trade) and qualitative data, for example,. quotations from sources featured in the article which state or assert something in relation to Pakistan’s illicit tobacco trade).

A further inclusion test (stage 2), consisting of coders manually checking the articles to determine if they included a claim and/or estimate about Pakistan’s ITT, was also conducted. Duplicate articles were also screened out during this process, though it should be noted that identical articles published by the same media outlet on different days were not considered duplicates. This approach ensures that findings from articles published multiple times throughout the studied period were not lost from our analysis.

### Triangulation With Urdu-Language Content

As our primary dataset is limited to English-language content, in order to triangulate our findings across local-language newspapers, we conducted additional searching and coding of a small sample of Urdu-language news articles. This involved applying the search term “غیر قانونی تمباکو”

(“illicit tobacco” in Urdu) in a popular internet search engine and filtering results to within 2015–2020. The first two results for each year that met the inclusion criteria were then used, creating a sample that captures articles from a variety of Urdu publications. More articles would have been included had more relevant results been obtained from the search process, but we found only a small number of relevant results in some years, resulting in two articles per year being the most viable sample we could use. These articles were then coded by authors proficient in Urdu and then double-coded by another author proficient in Urdu, with input from the lead author.

### Data Analysis

A coding framework was developed by AWAG and ZDS before being piloted on a random sample of five articles and then refined accordingly. The final framework (Table S2) focused on capturing basic information about the articles, data sources within the articles (Table 1), key narratives or arguments captured within the articles, and any policy recommendations featured within the articles. The framework captures both specific claims (eg, estimates of illicit trade) as well as wider claims (eg, content on drivers of illicit trade or policy recommendations).

**Table 1.** Source Types and Definitions

Source type	Description
Local government	Sourced from a quotation from a government representative or from a government report, for example, Ministry of Health, FBR.
Intergovernmental	Sourced from a representative or from a report by an intergovernmental organization, for example, OECD, UN, and WHO.
Academic	Sourced from a quotation by an academic or reference to a paper by a research group housed within an academic institution (eg, University of Bath).
Tobacco company/tobacco industry representative	Directly attributed to a tobacco company/a quotation from a tobacco industry representative.
Market research company	Sourced from a market research company website or report (eg, Euromonitor and Nielson).
Accountancy firm	Sourced from a quotation by a representative of a global accountancy firm or a report by such a firm (eg, KPMG and Deloitte).
Tobacco control advocacy organization	Sourced from a quotation by a representative of a tobacco control advocacy organization or a report by such a group, for example, Framework Convention Alliance.
Another third party	Sourced from a third party which does not meet any of the above, for example, Interpol, Oxford Economics, International Tax, and Investment Center.

A team of 7 then conducted the coding and 10% of the articles (35) were double coded (either by AWAG or ZDS) and tested for intercoder reliability. To calculate intercoder reliability, the double-coded data (captured in an Excel spreadsheet) was then submitted to the online utility ReCal2 (http://dfreelon.org/utils/recalfront/) which determines percent agreement, Scott’s pi, Cohen’s kappa, and nominal Krippendorff’s alpha. These are three commonly used specific statistics used to measure intercoder reliability for nominal-level data with two coders. While this project involved numerous coders, the coding data was treated as nominal to ensure that a clear, single final percentage could be identified at the end of the process.

Overall intercoder reliability between “initial coder” (an even sample of UA, MR, AU, SU, and HU coding) and “double coder” (17 coded by AWAG and 18 by ZDS) was conducted resulting in an average 88% agreement. Remaining discrepancies were then resolved through discussion between AWAG & ZDS. As AWAG & ZDS were the only coders also involved in the development of the coding framework, it was deemed appropriate that they conduct the double coding and agree with discrepancies between themselves to ensure the highest possible quality of the final coding. Similarly, for the Urdu-language coding MR, AU, and SU collectively coded the articles, which were then double-coded by ZDS, resulting in overall intercoder reliability average of 79.9%, with ZDS and AWAG then discussing the coding to resolve any discrepancies.

### Exploration of Media Coverage Over Time

To explore trends in media coverage over time in the lead-up to and directly following the annual budget announcements, additional analysis was conducted on the English-language sample. First, we identified the number of relevant articles 2 months prior to (April–May) and 2 months following (July–August) the annual budget announcement in the month of June each year, then we aggregated count data for April–May and compared with aggregated count data in July–August for each year before testing the statistical significance of any observed spikes in the data using the Wilcoxon signed-rank test. This test was deemed appropriate due to its suitability for non-parametric data, that is, data that does not rely on any specific assumptions about its parameters.^27^

To triangulate the findings from the Wilcoxon test, a Poisson Regression analysis was also conducted. This regression technique was chosen for its appropriateness in analyzing count data.^28^

We did not conduct similar analysis on the Urdu-language sample due to our internet searching only revealed a small amount of relevant articles for the studied years, meaning our sample size was too small to provide valuable insight into coverage on a monthly basis.

## Results

This section begins with an overview of the findings from the English-language content specifically, before exploring the Urdu-language content in the final sub-section.

### Overview of Publications

The initial filtered database search identified 544 articles, 216 of which were then excluded during the second inclusion stage, resulting in a final dataset of 328 articles (Figure S1).

Of the 22 news outlets identified in our English-language dataset, The News International appeared most frequently (19.5%) followed by daily times (12.1%) and Dawn (11.5%). Individual circulation figures for each newspaper were not available but seven are considered leading by virtue of being featured in Gallup’s monthly newspaper content analyses.^29^

### Overview of Publications Over Time

**Figure 1.**
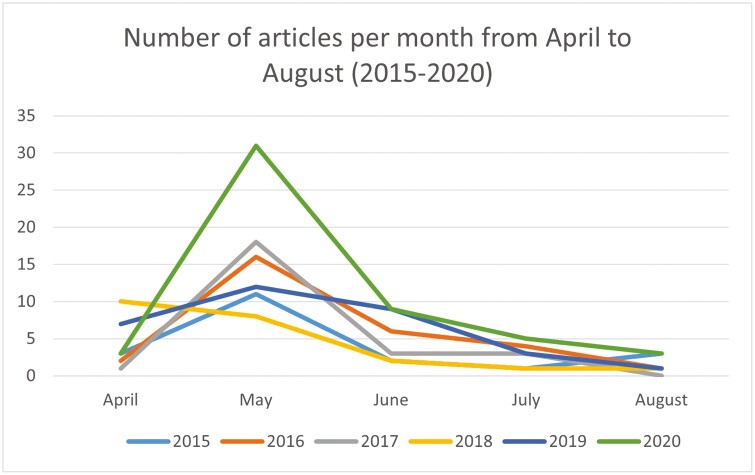
Number of articles per month from April to August (2015–2020). A graph depicting the number of relevant newspaper articles identified from April to August of each year, from 2015–2020. It shows that, for every year, May saw featured the highest number of relevant articles.

Understanding the number of articles over time can provide insight into when ITT has featured most often within newspaper coverage in Pakistan. Our findings indicate that, between 2015 and 2019, the number of articles relevant to ITT remained consistent between 45 and 50 articles per year. However, in 2020 the number of articles doubled. When breaking down frequency of relevant articles in the months leading up and directly following the June annual budget announcements, for all years, there was a spike in articles in the month prior to the budget (May; Figure 1).

A Wilcoxon signed-rank test on this data created a *p*-value of .03125, indicating a statistically significant difference in the frequency of relevant articles between the months of April and May, compared to the other months. Similarly, the Poisson regression model indicates that there is a statistically significant difference between the counts in April–May and July–August, as evidenced by the *p*-values for the coefficients being less than the conventional significance level of .05. Specifically, the coefficient for the treatment level “April–May” is statistically significant (*p* < .05), indicating that there is evidence of a difference in counts between April–May and July–August. The *p*-value for the LLR (Log-Likelihood Ratio) test is extremely low (*p* < .001), suggesting that the model significantly improves the fit compared to the null model.

### Sources of Data Featured in Articles

Three hundred and fifty-seven data points with identifiable sources were identified within the sample of 328 articles (Table S3; this is higher than the total number of articles due to some articles featuring multiple claims and the coding framework allowing for multiple claims to be coded individually). The most prevalent sources were national government bodies (36.6%), followed by tobacco companies or their representatives (15.1%), and then market research companies (13.1%).

Of third parties which did not fit neatly within the categories featured in the coding framework (9.5%), Oxford Economics and the International Tax and Investment Center (coded collectively due to being coauthors on various outputs throughout the period of research) appeared most often.

### Overview of Industry-Funded Content Featured Within Articles

Of the 357 identifiable claims/estimates featured in the coded articles, 66 (20%) were identified as industry-funded, either directly (16.4%), or indirectly (3.6%; Table 2).

**Table 2.** Overview of Industry-Funded Sources and Claims/Estimates Featured in Media Articles

directly funded claims	
Company name	Number of claims
British American Tobacco (BAT)/Pakistan Tobacco Company (PTC)	20
Philip Morris/Philip Morris Pakistan Limited (PMPKL/Philip Morris International [PMI])	22
Undisclosed tobacco company (article refers to a tobacco industry source but does not disclose it)	12
Total	54
*indirectly funded claims*	
Reports by International Tax and Investment Center/Oxford Economics (reports funded by Philip Morris and related subsidiaries)	11
Report by Nielsen (funded by PTC and PMPKL)	1
Total	12

Philip Morris and its subsidiaries were the most identified source of claims/estimates relating to Pakistan’s ITT (22), followed closely by British American Tobacco (BAT) and its subsidiary, Pakistan Tobacco Company (PTC; 20), with all other examples of direct funding coming from instances where the article acknowledged that the claim/estimate came from the tobacco industry, but did not disclose the relevant company (12).

Of the indirectly funded claims, almost all came from Oxford Economics and the International Tax and Investment Center (OE/ITIC; which have co-produced a number of industry-funded reports^30–32^), with the sole exception being a Nielsen report from 2015 which was funded by Philip Morris Pakistan and PTC.^33^

Of the 11 instances where OE/ITIC was the claim/estimate source, in only one of these were OE/ITIC’s funding relationship with the industry acknowledged in the article, meaning that in all other instances, OE/ITIC’s links to the industry were not disclosed when claims/estimates sourced from them were cited.

### Prevalence of Industry Funded Claims/Estimates

Figure S2 demonstrates the number of claims/estimates with an identifiable source which were featured in articles 2 months prior to and 2 months following the annual budget announcement (June) from 2015 to 2020. Overall, the findings demonstrate a clear spike in claims/estimates relating to Pakistan’s ITT in the month prior (May) to the budget announcement indicating a potential link between media discussion on ITT and the lead-up to the annual budget announcement.

### Overview of Articles Containing Claims/Estimates

The most common claim/estimate featured across the dataset was estimates of government revenue loss because of ITT (42.3%), followed by estimates of Pakistan’s ITT (36.5%), and references to the federal budget discussions (19.2%).

(Table S1). The type of claims appearing in media articles were comparable across articles citing both industry and non-industry sources.

While Table S1 provides insight into the overall prevalence of tobacco industry sources being cited in relation to specific claims/estimates it lacks nuance in relation to the nature of these claims/estimates and how these may differ between sources. For instance, 23 articles included claims that ITT rose or was likely to rise because of tobacco control policy, and 9 of these articles included industry-sourced data, but this provides limited input into the policies in question, and any nuances between the sources making such claims. As such, additional qualitative insight for a selection of the claims/estimates that the authors felt would add most value to the analysis is also included in Supplementary Material.

### Overview of Urdu-Language Content

Urdu-language content from five different publications featured in our sample of 12 articles (two articles per year from 2015 to 2020), with Nawa-i-Waqt being the most prevalent. The top claims/estimates featured in the articles were claims of revenue loss resulting from ITT (66.7%), estimates of Pakistan’s ITT (50%), claims that Pakistan has the highest/one of the highest rates of ITT in Asia (25%), and claims that Pakistan’s ITT had risen in recent years (25%; Table S4).

When comparing these findings to the English-language sample, the results are broadly similar, with the two most frequent claims/estimates featured in the Urdu sample being the same as for the English sample and with most cited source type also being national government (50%). As was the case with the English-language sample, ITIC/OE estimates, which are industry-funded, were cited in several Urdu articles without any disclosure that the cited data originated from an industry-funded source.

## Discussion

Our findings demonstrate that there was a statistically significant increase in the number of English-language articles featuring claims/estimates relating to Pakistan’s ITT in the months leading up to the annual budget each year. The most prevalent data sources in articles relating to Pakistan’s ITT, were national government bodies, followed by tobacco companies.

This is likely a reflection of journalistic standards, whereby official government sources are prioritized over others and so journalists actively seek them for their stories, even if the government is less active in producing relevant data than the tobacco industry. However, such standards can also involve presenting “both sides” of a debate, which can lead to issues being presented as more controversial/debated than is actually the case.^34^ This may go some way in explaining why tobacco industry sources (15.1%) were the second most common in the dataset while also explaining the large number of tobacco control advocacy organizations featured (with articles often citing both to provide differing viewpoints on the same issue). However, it should also be noted that, within the context of Pakistan, not all government sources are equal in terms of independence from the tobacco industry. For instance, seven of the government-sourced claims/estimates identified in the dataset came from the Pakistan Tobacco Board—a Government body set up to promote tobacco cultivation and trade.^35^

Our finding that the types of claims/estimates appearing in media articles were comparable across articles citing both industry and non-industry sources indicates that the individual aims and focus of journalists when writing stories appear to be the main factor in what topic areas the story covers, rather than the data cited. While this does not mitigate the impact of unreliable data being spread within media articles, it does demonstrate that manipulation of data alone may not always be sufficient to manipulate wider media narratives. This conclusion is supported by the prominence of articles, which were critical of both national and international tobacco companies in relation to their potential involvement in ITT, indicating that TI company’s efforts to present themselves as solutions to ITT, seen in countries across the world,^36,37^ do not appear to have successfully transferred to media conversations around ITT in Pakistan.

Our findings also identify several areas of concern regarding media coverage of ITT in Pakistan and how TI-funded claims are used within it. While small in proportion to the overall dataset, it should not be overlooked that there were multiple instances of industry-funded claims/estimates being cited in media coverage without their links to industry being disclosed and that the total number of such cases are likely underreported in our findings, given the difficulties in identifying indirect industry funding within the often-limited context provided by media articles. For instance, while only one datum could be linked to the industry-funded Nielsen report, it is highly likely that number of other claims also came from this same source, meaning that our findings likely underrepresent the number of times tobacco industry claims/estimates were indirectly included in media articles.

The report was released in September 2015 and 11 articles from the dataset cited Nielsen in the month following the release of the report but without providing enough detail for coders to identify if the Nielsen claim/estimate being cited stemmed from the industry-funded 2015 report. It is thus entirely possible that these were also referencing industry funding claims/estimates without disclosing this. Forty claims/estimates in total across the English dataset, and two from the Urdu sample, were attributed to Nielsen so it is possible that the number of indirectly industry-funded and unacknowledged claims/estimates featured in the dataset is higher still.

Similarly, 18% of the claims/estimates identified as being industry-funded were claims where the tobacco industry was cited but without naming a specific company, demonstrating that there are transparency issues in relation to Pakistani media coverage of ITT, and this was reflected also in the Urdu sample. These complexities are perhaps unsurprising, given prior evidence that the tobacco industry is reliant on increasingly complex webs of third parties to propagate its narratives, including those relating to ITT.^37,38^

### Policy Implications

Our findings identify several weaknesses within media coverage of ITT in Pakistan which leave the door open for the tobacco industry’s efforts to influence policy within the country. While not unique to Pakistan, nor simple to address, the government may wish to consider new initiatives and standards to improve the transparency and thus overall quality of media coverage to address such concerns. Journalists could be encouraged to not cite unnamed tobacco industry sources, and to provide disclaimers on the funding sources of the reports they cite, for instance.

The government should be cautious of perceiving media narratives as being accurate reflections of the public’s position on topics such as TT and resist any industry efforts to use media coverage to pressure the government and attempt to influence its decision-making. One finding from our work is that national government authorities were the primary source of claims/estimates relating to Pakistan’s ITT, demonstrating that the government is already pro-actively engaging with media in the country and such efforts should be continued to counter questionable tobacco industry claims/estimates from becoming the norm. Of note is that tobacco control advocacy organizations also featured prominently in the dataset, demonstrating that there is a strong and well-established tobacco control community in Pakistan which likely also plays a role in preventing the tobacco industry from becoming the dominant voice in media discussions regarding ITT.

Nonetheless, the most effective strategy for ensuring accurate understandings of ITT is for the government to draw from independent sources for monitoring and estimating the country’s ITT. Furthermore, the government could utilize the country’s track and trace system, mandated in 2022, as a means of identifying ITT, with the system serving as a regular data source. However, it is essential that the country’s system is protected from tobacco industry interference, particularly given that such interference was previously documented in Pakistan.^39–42^

Additional policy measures to address ITT should also be considered. Pakistan ratified the FCTC Protocol to Eliminate Illicit Trade in Tobacco Products in 2018 which contains several measures beyond the introduction of a tracking and tracing system, such as requiring Parties to implement effective controls of tobacco products within free zones, or special economic zones, as they are known in Pakistan. Further, as noted in the World Bank’s report on country experiences in addressing ITT, key drivers of the practice include weaknesses in governance and the regulatory framework, corruption, insufficient capacity of enforcement and judiciary systems, the existence of informal distribution, and organized crime networks, among others.^2^

Evidence suggests that on many of these fronts, there is further room for improvement in Pakistan. A 2021 study measured the capacity of 160 countries to combat ITT, gave Pakistan a final score of 136 out of the 160 countries studied (the smaller the number, the more effectively a country is placed to address ITT).^7^ In Transparency International’s Corruption Index, Pakistan ranked 124th most corrupt (tied with several other countries) out of the 180 countries considered.^43^ Looking at tobacco industry interference as a factor specifically, The Global Tobacco Industry Interference Index of 2023, which ranks countries by how much tobacco industry interference occurs within them, indicated that industry interference is worsening in the country. As such, the government of Pakistan’s response to ITT will require solutions beyond measures to solely address ITT, but also to address wider problems in the country, such as corruption, organized crime, and tobacco industry policy interference, among others. Strengthening governance and law enforcement in the country will also serve to improve the likelihood of the track and trace system having the desired impact of reducing ITT.

### Strengths and Limitations

Given the detailed nature of this work’s quantitative presentation of a substantial amount of qualitative data, its key strength lies in the breadth of data that it captures. Our findings cover a 5-year period and a broad scope capturing = any claim/estimate relating to Pakistan’s ITT within all major English-language publications in the country. We also utilized a small sample of Urdu newspaper articles to triangulate our findings across several languages. We hope that the approach outlined in this work can be replicated in other countries so that the underexplored topic of tobacco industry’s influence over media coverage on ITT can become a more common focus of research going forward, particularly within LMIC contexts. To our knowledge, our framework is the first to systematically extract media narratives about ITT from media articles, although it does draw from approaches adopted in prior work which extracted data on ITT from other sources.^6^

Nonetheless, there are several limitations to this work. Firstly, given the 5 year period and multiple newspapers covered in our study, we utilized a targeted search strand which names individual tobacco companies in order to create a manageable sample. This creates a risk of introducing bias whereby our data may overemphasize the proportion of articles citing industry data within Pakistan’s overall media landscape. Secondly, the data could also be an underestimate of the problem as the LexisNexis database does not capture newspaper advertisements, meaning industry-funded newspaper campaigns are overlooked in the dataset despite these likely providing insight into the influence of the tobacco industry within Pakistan’s media. Thirdly, our dataset was largely limited to English-language publications, limiting the generalizability of our findings. However, English is a co-official language of Pakistan, spoken by 49%–58% of the population, and so is still an appropriate language for this analysis and our inclusion of 12 Urdu-language articles to triangulate our findings indicates that they are unlikely to be unique to English-language publications. It should be noted, however, that the Urdu-language searching provided far less results than was the case with the English-language searching. This may indicate that Urdu-language media outlets less frequently cite industry data on ITT and feature less discussion on ITT more broadly.

Fourthly, while efforts were made to make our framework as comprehensive as possible whilst still accessible and practical for coders to use, it is nonetheless subject to some limitations. For instance, the framework does not attempt to capture any country-specific narratives related to ITT which may be prevalent in Pakistan. This is another gap that other research may wish to explore, given prior evidence that the industry frequently uses country-specific narratives in order to try and influence tobacco tax policy outcomes.^20^ Furthermore, our coding was somewhat streamlined in that our focus was on identifiable claims/estimates, thus excluding claims where no indication of the source was provided whatsoever (a common occurrence). We also did not explore “the source behind the source” thus limiting the depth of our data for example, there may be instances where a government official is citing industry-funded data but without this being disclosed, and our framework would miss this.

## Supplementary material

Supplementary material is available at *Nicotine and Tobacco Research* online.

ntae133_suppl_Supplementary_Materials

## Data Availability

The data underlying this article will be shared on reasonable request to the corresponding author.
