# Supplementary material for: A Systematic Investigation of Tobacco Industry Sourced Data Relating to Illicit Tobacco Trade Featured in Pakistan’s Media Coverage (2015–2020)
Source: Nicotine Tob Res. 2024 May 31;26(11):1489–96. doi: 10.1093/ntr/ntae133 (PMC11494476; doi:10.1093/ntr/ntae133)
Supplement: ntae133_suppl_Supplementary_Materials [file ntae133_suppl_supplementary_materials.docx]

**A systematic investigation of tobacco industry sourced data relating to illicit tobacco trade featured in Pakistan’s media coverage (2015-2020): Supplementary Appendix**

**Supplementary Figure 1: PRISMA Diagram (for English-language articles)**

**Identification of studies via databases and registers**

Records removed *before screening*:

Duplicate records removed (n = 0)

Records marked as ineligible by automation tools (n = 0)

Records removed for other reasons (n = 0)

Records* identified from:

Databases (LexisNexis) (n = 544)

Registers (n = 0)

**Identification**

Records excluded**

Duplicate (n = 64)

Records screened

(n = 544)

**Screening**

Records excluded:

Do not meet inclusion criteria (n = 152)

Records assessed for eligibility

(n =480)

Records included in review

(n = 328)

**Included**

*As our data consists solely of newspaper articles, we have modified the standard PRISMA diagram template to focus solely on ‘records’ rather than records, reports, or studies as these additional categories are not applicable to our dataset. As such, each record reported here is a newspaper article.

*Adapted From:*  Page MJ, McKenzie JE, Bossuyt PM, Boutron I, Hoffmann TC, Mulrow CD, et al. The PRISMA 2020 statement: an updated guideline for reporting systematic reviews. BMJ 2021;372:n71. doi: 10.1136/bmj.n71

**Supplementary Figure 2: Funding status of identifiable sources of claims/estimates within articles 2 months before and after budget announcement (2015-2020)**

**
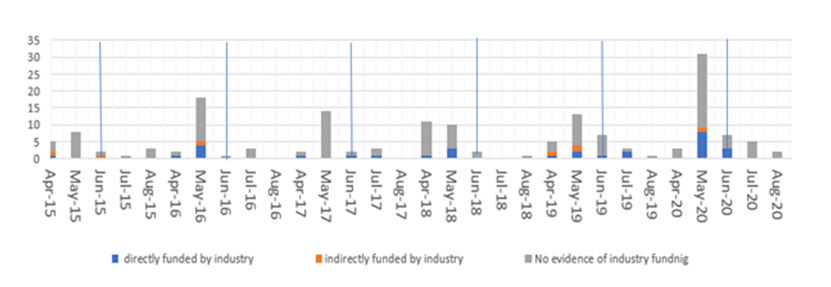
**

| **Supplementary Table 1: Nature and number of claims/estimates points and articles containing them** | | | |
| --- | --- | --- | --- |
| **Types of claims/estimates captured from articles** | **Description / definition / example** | **Number and % of articles claim/estimates featured in** | **Number and % of these which contain data from industry-funded sources** |
| Claim of revenue loss to Pakistan's government as a result of illicit tobacco trade | eg. 'the illicit cigarette trade deals an estimated loss of Rs24 billion per annum to the national exchequer'. | 139 (42.3%) | 30 (21.5%) |
| Estimate of Pakistan's ITT | Estimate= a calculation or judgement of the value, number, quantity, or extent of something.    Illicit tobacco trade= The production, import, export, purchase, sale, or possession of tobacco goods which fail to comply with legislation. | 120 (36.5%) | 29 (24.1%) |
| Reference to federal budget discussions | The Federal budget= the federal budget of Pakistan for each fiscal year beginning from 1 July each year and ending on 30 June 2020. Article may refer to the upcoming federal budget, or to a previous federal budget, or may refer to government discussions about the federal budget. Mentions of the Government's revenue/spending alone are not included here. | 63 (19.2%) | 13 (20.6%) |
| Claim that Pakistan's illicit tobacco trade had risen in recent years? | Recent = 2015-2020. | 51 (15.5%) | 11 (21.5%) |
| Claim that the tobacco industry is a contributor/facilitator of illicit tobacco trade | Contributor/facilitator = contributes or knowingly facilitates illicit tobacco trade. Claim does not need to be specific to Pakistan's illicit tobacco trade. | 50 (15.2%) | 13 (26%) |
| Claim that Pakistan's illicit tobacco trade rose, or was likely to rise, as a result of a tobacco control policy | Article mentions that a tobacco control policy, either proposed or already in force, has or would if enforced, increase/d Pakistan's illicit tobacco trade. | 23 (7%) | 9 (39.1%) |
| Critique of claim/estimate featured in article | critique= analyse and judge the claim/estimate on its merits and faults/ identifying strengths or weaknesses. | 23 (7%) | 6 (26%) |
| A reference to the Protocol | The Protocol: the World Health Organisation, Framework Convention on Tobacco Control, Protocol to eliminate illicit trade in tobacco products. | 25 (7.6%) | 3 (12%) |
| Claim that Pakistan has the highest/one of the highest rates of illicit tobacco trade in Asia | Article uses specific language comparing the size of Pakistan's illicit tobacco trade with other countries in Asia. | 22 (6.7%) | 8 (36.3%) |
| Claim that the tobacco industry is part of the solution to address illicit tobacco trade | Part of the solution = actively works to reduce illicit tobacco trade and/or supports measures to reduce it. | 2 (0.6%) | 1 (50%) |
| A policy recommendation from a tobacco industry representative | Policy recommendation = policy (a principle or action) advice for a group that has the authority to make decisions eg. the government. | 4 (1.2%) | 4 (100%) |

| **Supplementary Table 2: Coding framework overview** | |
| --- | --- |
| **Information captured** | **Key definitions / descriptions** |
| Date of publication | Date the article was published. |
| Name of newspaper | Name of the media outlet which published the article. |
| Name of author/journalist (if disclosed) | Name of the person the article is credited to. |
| Claim/estimate source type | See Table 2 (claim/estimate source types). |
| Name of source | Name of the individual or organisation that the claim is attributed to. |
| Evidence of source being tobacco industry-funded* | Industry-funded: has been funded fully, or in part, by tobacco companies including industry-produced research and quotations from industry representatives (direct) and research commissioned by industry but conducted by other companies such as market research organisations (indirect). |
| Does article disclose that any claims/estimates in it are industry-funded? | Does the article disclose that any of the claims/estimates featured within it are industry funded? Eg. identifying a specific tobacco company, or acknowledging that a tobacco company contributed to the funding of a specific piece of information. |
| What years (if any) does the claim/estimate refer to? | If a claim/estimate applies to a specific time period which is mentioned in the article eg. Pakistan’s ITT in 2020. |
| Does the article include an estimate of Pakistan’s ITT? | eg. % of market that is illicit) and/or an estimate of the makeup of the illicit market (eg. % of specific type of products such as counterfeits, etc |
| Does the article include an estimate on Pakistan’s government revenue loss as a result of the ITT? | Does the article provide a calculation or judgement of the extent of revenue loss to the Pakistan government as a result of the ITT? |
| Is it claimed that Pakistan has the highest/one of the highest rates of ITT in Asia? | This question aims to capture a single very specific claim- that Pakistan has the highest, or one of the highest, rates of ITT in Asia. |
| Is Pakistan’s ITT said to have risen in recent years? | This question asks if the articles make any references to Pakistan’s ITT growing in recent years (2015-2020). |
| Is Pakistan’s ITT said to have risen, or likely to rise, as a result of a tobacco control policy? | This question considers if articles mention that a tobacco control policy (this could be a policy introduced in Pakistan or elsewhere) has impacted Pakistan’s ITT. |
| Are tobacco companies presented as a solution to or contributor to illicit trade? | Solution = described in article as working to address illicit tobacco / are part of the solution / article outlines examples of industry initiatives to counter ITT (historical or contemporary).  Facilitator/contributor = described in article as part of the problem of ITT / article outlines examples of industry involvement in ITT (historical or contemporary). |
| Are any other drivers of illicit trade mentioned in the article? | Mention of any other factors which are said to increase/drive Pakistan’s ITT. Eg. government corruption, border density. |
| Does the article feature critique any of the claims/estimates in Pakistan that it mentions? | critique= analyse and judge the data on their merits and faults/ identifying strengths or weaknesses of data. |
| Is there a reference to federal budget discussions? | The Federal budget= the federal budget of Pakistan for each fiscal year beginning from 1 July each year and ending on 30 June. Article may refer to the upcoming federal budget, or to a previous federal budget, or may refer to government discussions about the federal budget. Mentions of Government's revenue/spending alone are not captured here. |
| Is a policy recommendation from a tobacco company or representative is featured? | Policy recommendation = policy (a principle or action) advice for a group that has the authority to make decisions eg. the government. |
| Include any other policy recommendations related to illicit trade? | This questions captured policy recommendations from non-industry sources. |
| Does the article mention the Protocol to Eliminate Illicit Trade in Tobacco Products? | This question captures any mention of the Protocol within the article, within any context. |

*Industry funding was determined via investigative analysis (following an approach identified in previous research^34 35^) of the source of the claim/estimate as well as efforts to identify the source material of the claim/article (eg. a specific report) and searching that material for financial disclosures.

| **Supplementary Table 3: Sources featured in articles (English)** | | |
| --- | --- | --- |
| **Source type** | **Definition** | **Number (% of total)** |
| Total number of identifiable claims | Total number of relevant claims identified in articles | 357 (100%) |
| Academic | The source is an academic or a research paper by a research group housed within an academic institution. (eg. Johns Hopkins Bloomberg School of Public Health, University of Illinois, Chicago). | 7 (1.9%) |
| Accountancy firm | The source is a report by or a representative of a global accountancy firm (eg. KPMG, Deloitte). | 3 (0.8%) |
| Intergovernmental | The source is report by, or representative from, and intergovernmental organisation (eg. WHO, UN). | 29 (8.1%) |
| Market research company | The source is a company which specialises in producing market research (eg. Euromonitor, Nielsen). | 47 (13.1%) |
| National government | The source is a report by a national government organisation or is a representative of such an organisation (eg. Ministry of Health, FBR). | 131 (36.6%) |
| Tobacco company or industry representative | The source is a tobacco company or a tobacco company representative (eg. BAT, PMI). | 54 (15.1%) |
| Tobacco control advocacy organisation | The source is a tobacco control advocacy organisation or representative of such an organization. (eg. Framework Convention Alliance, Pakistan National Heart Association). | 39 (10.9%) |
| Other third party (parties listed below): | The source is a third party not deemed to meet any of the above criteria. | 34 (9.5%) |
|  | **Number** |  |
| Fikr e-Fardan Organisation | **2** |  |
| Human Development Foundation (HDF) | **7** |  |
| Network for Consumer Protection, The (Pakistan) | **8** |  |
| Overseas International Chamber of Commerce and Industries (OICCI) | **1** |  |
| Oxford Economics/International Tax and Investment Centre (ITIC)* | **10** |  |
| People’s Lawyer’s Forum | **1** |  |
| People’s Students’ Federation | **1** |  |
| Social Policy and Development Centre (SPDC) | **4** |  |

*The International Tax and Investment Centre and Oxford Economics have produced multiple reports on illicit tobacco trade which were funded by TTCs.^14 15^

| **Supplementary Table 4: Nature and number of claims/estimates and articles containing them (Urdu)** | | |
| --- | --- | --- |
| **Types of claims/estimates captured from articles** | **Number of articles featured in** | **Number and % of these which contain data from industry-funded sources** |
| Claim of revenue loss to Pakistan's government as a result of illicit tobacco trade | 8 (66.7%) | 2 (25%) |
| Estimate of Pakistan's ITT | 6 (50%) | 2 (33.3%) |
| Claim that Pakistan has the highest/one of the highest rates of illicit tobacco trade in Asia | 3 (25%) | 2 (66.7%) |
| Claim that Pakistan's illicit tobacco trade had risen in recent years? | 3 (25%) | 1 (33.3%) |
| Claim that Pakistan's illicit tobacco trade rose, or was likely to rise, as a result of a tobacco control policy | 2 (16.7%) | 0 (%) |
| Reference to federal budget discussions | 2 (16.7%) | 0 (0%) |
| Claim that the tobacco industry is a contributor/facilitator of illicit tobacco trade | 0 (0%) | 0 (0%) |
| Critique of claim/estimate featured in article | 0 (%) | 0 (%) |
| A reference to the Protocol | 0 (%) | 0 (%) |
| Claim that the tobacco industry is part of the solution to address illicit tobacco trade | 0 (%) | 0 (%) |
| A policy recommendation from a tobacco industry representative | 0 (%) | 0 (%) |

**Additional qualitive insight**

**Policy recommendations and opposition to tobacco control policies**

Direct policy recommendations from tobacco industry representatives were limited within the dataset and were general in nature, for instance “PTC officials” requesting the government “to drive a balanced and evidence-based fiscal agenda throughout the year along with effective enforcement measures so as to curtail the sale of tax-evaded cigarettes”.^1^ Policy recommendations from other sources were more common and detailed, for instance making specific mention of implementation of a track and trace system ^2^ as well full implementation of the Protocol to Eliminate Illicit Trade in Tobacco Products as a whole^3^ increased tobacco taxation,^4^ 24/7 monitoring of a tobacco thrashing facilities,^5^ among others.

While limited direct policy recommendations from tobacco industry representatives were identified in the dataset, there were various examples of clear instances of tobacco companies attempting to use the threat of an increased ITT to oppose potential tobacco control measures. For example, one article from 2015 referred to a letter from a “top official” within PTC to the FBR which warned the tax authority that against increasing the size of pictorial health warnings on cigarette packs, arguing that such warnings would lead to an “increase in sale of smuggled cigarettes”.^6^ Similarly, an article from that later same year stated that “Experts” told the publication that “shifting from 40 percent to 85 percent pictorial warning will increase illegal supply of cigarettes”.^7^ As of March 2023, an 85% pictorial warning has not been introduced in Pakistan.

Critical references were also made to the government’s minimum price laws, with an article from 2019 summarising a PTC official as saying that the then-minimum of Rs 49.39 per pack was so much higher than the Rs15-Rs30 per pack of the locally manufactured illicit products that the policy creates “an uneven playing field for legitimate manufacturers”.^1^

Instances of tobacco industry efforts to use the ITT as a narrative to achieve industry-favourable policy outcomes were also identified within media discourses, for instance a 2017 “Dawn (Pakistan)” article, which refers to claims by the then-CEO of The Network for Consumer Protection (Pakistan) that “the international tobacco industry had created a wrong impression through deceptive statistics that the counterfeit tobacco industry has captured the market of the international tobacco companies, therefore FBR (Federal Board of Revenue) instead of raising tax, should lower it so that the international industry could compete with counterfeit cigarettes”.^8^

In addition to such examples of industry representatives using the ITT as an argument against tobacco control policy measures, similar arguments were also identified by sources not directly linked to the industry, for instance, a 2016 “The News International” article claiming that “the demand for illegal cigarettes is also rising because consumers do not want to carry local packs with horrible health warnings printed on them. Rather, they prefer packs, which carry no health warning at all. Adding that there is no dearth of such smuggled cigarette packs in shops and markets across the country”^9^. A 2020 “Pakistan Today” article states that “further taxation would… result in a repetitive increase in the tax on cigarettes… in fact the illicit trade of cigarettes in the country could rise.”^1^

**Tobacco industry as contributor or facilitator**

Content relating to the tobacco industry being a contributor to ITT focussed on both local manufacturers as well as the broader tobacco industry/transnational tobacco companies. Mentions of local manufacturers included claims ranger from them providing “wrong data to FBR to evade taxes”,^10^ to “openly violating the health regulations”.^11^

Broader mentions of the tobacco industry claimed it is said to engage in activities such as giving the “impression as if smoking is an innocuous habit and is in danger because of illicit cigarette trade”,^12^ with *“big tobacco companies” keeping some of their production “undeclared to evade taxes while blaming presence of illicit cigarettes in the market”,^13^ and that* “big tobacco companies are allegedly involved in the promotion” of ITT.^14^ In the case of complicity in ITT of both references to local manufacturers and the broader tobacco industry, most sources cited were tobacco control advocacy organisations, usually in the form of direct quotations from their representatives.

1. Paracha S. 'Tax-evading tobacco companies only paid Rs1.5bn in FY18': Pakistan Today; 8 April 2019 [Available from: 'Tax-evading tobacco companies only paid Rs1.5bn in FY18' - Profit by Pakistan Today accessed 2023 28 January.

2. The News International. Counterfeit cigarettes, 29 October 2020.

3. The Nation. 'Tobacco leading cause of communicable disease outbreak', 14 February 2019.

4. The Nation. End to foreign tobacco industry data use sought, 24 January 2017.

5. The Nation. Finding the bottleneck, 11 June 2020.

6. Daily Times. The Network contends contents of tobacco company's letter, 27 February 2015.

7. Pakistan Observer. NHSRC poised to enact controversial cigarette warning label, 5 May 2015.

8. Dawn (Pakistan). 'Tobacco companies making profit at the cost of public health', 11 May 2017.

9. The News International. Up in smoke, 8 May 2016.

10. Times D. Cigarette smuggling causing over Rs 7b loss annually 5 April 2016 [Available from: <https://www.ficcicascade.in/cigarette-smuggling-causing-over-rs-7b-loss-annually/> accessed 10 March 2023.

11. The News International. Local cigarette manufacturers ‘burning’ rules, regulations with impunity 7 April 2019 [Available from: <https://www.thenews.com.pk/print/454497-local-cigarette-manufacturers-burning-rules-regulations-with-impunity> accessed 10 March 2023.

12. Pakistan Observer. CTC calls for strong policy to expose tobacco industry’s tactics 31 May 2020 [Available from: <https://pakobserver.net/ctc-calls-for-strong-policy-to-expose-tobacco-industrys-tactics/> accessed 10 March 2023.

13. The News International. Increased cigarettes’ sale to prove health disaster 27 November 2020 [Available from: <https://www.thenews.com.pk/print/749902-increased-cigarettes-sale-to-prove-health-disaster> accessed 15 March 2023.

14. Ahmadini A. Experts call for further curbs on tobacco industry: Pakistan Today; 13 May 2020 [Available from: <https://profit.pakistantoday.com.pk/2020/05/13/experts-call-for-further-curbs-on-tobacco-industry/> accessed 13 March 2023.
